# *Copynumber:* Efficient algorithms for single- and multi-track copy number segmentation

**DOI:** 10.1186/1471-2164-13-591

**Published:** 2012-11-04

**Authors:** Gro Nilsen, Knut Liestøl, Peter Van Loo, Hans Kristian Moen Vollan, Marianne B Eide, Oscar M Rueda, Suet-Feung Chin, Roslin Russell, Lars O Baumbusch, Carlos Caldas, Anne-Lise Børresen-Dale, Ole Christian Lingjærde

**Affiliations:** 1 Biomedical Informatics, Dept of Informatics, University of Oslo, Oslo, Norway; 2 Centre for Cancer Biomedicine, University of Oslo, Oslo, Norway; 3 Cancer Genome Project, Wellcome Trust Sanger Institute, Hinxton, Cambridge, UK; 4 Dept of Human Genetics, VIB and University of Leuven, Leuven, Belgium; 5 Dept of Genetics, Institute for Cancer Research, Oslo University Hospital Radiumhospitalet, Oslo, Norway; 6 Institute of Clinical Medicine, Faculty of Medicine, University of Oslo, Oslo, Norway; 7 Dept of Oncology, Division of Cancer, Surgery and Transplantation, Oslo University Hospital Radiumhospitalet, Oslo, Norway; 8 Dept of Immunology, Institute for Cancer Research, Oslo University Hospital Radiumhospitalet, Oslo, Norway; 9 Breast Cancer Functional Genomics, Cancer Research UK Cambridge Research Institute and Dept of Oncology, University of Cambridge, Li Ka-Shing Centre, Cambridge, UK; 10 Cambridge Breast Unit, Addenbrookes Hospital and Cambridge National Institute for Health Research Biomedical Research Centre, Cambridge University Hospitals NHS Foundation Trust, Cambridge, UK

**Keywords:** Copy number, aCGH, Segmentation, Allele-specific segmentation, Penalized regression, Least squares, Bioconductor

## Abstract

**Background:**

Cancer progression is associated with genomic instability and an accumulation of gains and losses of DNA. The growing variety of tools for measuring genomic copy numbers, including various types of array-CGH, SNP arrays and high-throughput sequencing, calls for a coherent framework offering unified and consistent handling of single- and multi-track segmentation problems. In addition, there is a demand for highly computationally efficient segmentation algorithms, due to the emergence of very high density scans of copy number.

**Results:**

A comprehensive Bioconductor package for copy number analysis is presented. The package offers a unified framework for single sample, multi-sample and multi-track segmentation and is based on statistically sound penalized least squares principles. Conditional on the number of breakpoints, the estimates are optimal in the least squares sense. A novel and computationally highly efficient algorithm is proposed that utilizes vector-based operations in R. Three case studies are presented.

**Conclusions:**

The R package copynumber is a software suite for segmentation of single- and multi-track copy number data using algorithms based on coherent least squares principles.

## Background

In cancer, the path from normal to malignant cell involves multiple genomic alterations including losses and gains of genomic DNA. A long series of studies have demonstrated the biological and clinical relevance of studying such genomic alterations (see, e.g., [1,2] and references therein). Genome-wide scans of copy number alterations may be obtained with array-based comparative genomic hybridization (aCGH), SNP arrays and high-throughput sequencing (HTS). After proper normalization and transformation of the raw signal intensities obtained from such technologies, the next step is usually to perform segmentation to identify regions of constant copy number. Many segmentation algorithms are designed to analyse samples individually (see, e.g., [3-16] and references therein), while most studies involve multiple samples, multiple tracks, or both. Joint handling of multiple samples is computationally and conceptually challenging, see e.g. [17,18]. Most systematic approaches for this problem are based on individual segmentation of each sample followed by post-processing to combine results across samples (see, e.g., [18] and references therein), while some recent publications propose strategies for joint segmentation of all samples [19-23]. Recently, the emergence of new technologies have pushed the limit of genomic resolution, opening new vistas for studying very short aberrations, including aberrations affecting only part of a gene or gene regulatory sites in the DNA. A major challenge raised by these novel technologies is the steadily growing length of the data tracks, which drastically increases the demand for computationally efficient algorithms. The occurrence of extreme observations (outliers) of biological or technical origin pose an additional challenge, as most segmentation methods are substantially affected by such observations. Picard et al. [6] propose a least squares based segmentation method that results in a piecewise constant fit to the copy number data. Their approach assumes that the user either supplies the desired number of segments or leaves to the method to automatically determine this number. In this paper, we describe a related approach. In particular, the proposed method utilizes penalized least squares regression to determine a piecewise constant fit to the data. Introducing a fixed penalty *γ*>0 for any difference in the fitted values of two neighboring observations induces an optimal solution of particular relevance to copy number data: a piecewise constant curve fully determined by the breakpoints and the average copy number values on each segment. The user defined penalty *γ*essentially controls the level of empirical evidence required to introduce a breakpoint. Given the number of breakpoints, the solution will be optimal in terms of least squares error.

To achieve high processing efficiency, dynamic programming is used (see [24]). To further increase computational efficiency, a novel vector based algorithm is proposed, and even further speed optimization is obtained through heuristics. A central aim of the present work has been to provide methodology and high-performance algorithms for solving single- and multiple-track problems within a statistically and computationally unified framework. All proposed algorithms are embedded in a comprehensive software suite for copy number segmentation and visualization, available as the Bioconductor package copynumber. Main features of the package include: 

• Independent as well as joint segmentation of multiple samples

• Segmentation of allele-specific SNP array data

• Preprocessing tools for outlier detection and handling, and missing value imputation.

• Visualization tools

## Implementation

### Systems overview

**Figure 1:**
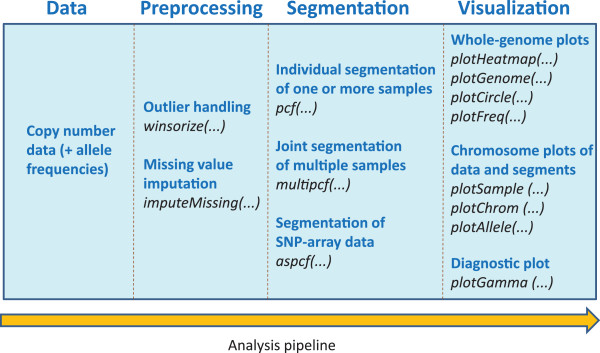
**An overview of the copynumber package.** Depending on the aim of the analysis, the input will be copy number data and possibly allele frequencies from one or more experiments. Preprocessing tools are available for outlier handling and missing data imputation, and three different methods handle single sample, multi-sample and allele-specific segmentation. Several options are also available for the graphical visualization of data and segmentation results.

The copynumber package provides functionality for many of the tasks typically encountered in copy number analysis: data preprocessing tools, segmentation methods for various analysis scenarios, and visualization tools. Figure 1 shows an overview of the typical work flow. Input is normalized and log_2_-transformed copy number measurements from one or more aCGH, SNP-array or HTS experiments. Allele-frequencies may also be specified for the segmentation of SNP-array data. It is strongly recommended to detect and appropriately modify extreme observations (outliers) prior to segmentation, as these can have a substantial negative effect on the analysis. For this purpose, a specially designed Winsorization method is included in the software package. A missing-value imputation method appropriate for copy number data is also available.

Segmentation methods for three different scenarios (single sample, multi-sample and allele-specific segmentation) are implemented in the package. All these methods are referred to as Piecewise Constant Fitting (PCF) algorithms and seek to minimize a penalized least squares criterion. In single sample PCF, individual segmentation curves are fitted to each sample. In multi-sample PCF, segmentation curves with common segment borders are simultaneously fitted to all samples. In allele-specific PCF, the segmentation curves are fitted to bivariate SNP-array data, providing identical segment borders for both data tracks. A set of graphical tools are also available in the package to visualize data and segmentation results, and to plot aberration frequencies and heatmaps. Also included are diagnostics to explore different trade-offs between goodness-of-fit and parsimony in terms of the number of segments. In the remaining part of this section, a formal description of the algorithms is given. However, note that these details are not a prerequisite for reading later sections or for using the copynumber package.

### Preprocessing: Outlier handling

A challenging factor in copy number analysis is the frequent occurrence of outliers - single probe values that differ markedly from their neighbors. Such extreme observations can be due to the presence of very short segments of DNA with deviant copy numbers, to technical aberrations, or a combination. When identification of CNVs is a purpose of the study, the multi-sample method described below may be applied for such detection. However, when the focus is on detection of broader aberrations, the potentially harmful effect of extreme observations on aberration detection methods induces a need for outlier handling procedures (see, e.g., [3,6]). Since the copynumber package is based on least squares, an extreme observation will tend to cause the detection of a short segment. When searching for broader segments, such short (and abundant) segments will represent noise and may also affect the identification of other segments. We therefore now describe a procedure for reducing the effect of extreme observations, while the effects of this method will be considered in the Results and discussion section. Winsorization is a simple transformation reducing the influence of outliers by moving observations outside a certain fractile in the distribution to that fractile (see [25]). For identically distributed observations *y*_1_,…,*y*_*p*_, the corresponding Winsorized observations are defined as $y_{j}^{w}=\Psi (y_{j})$ where

$$\Psi (y)=\Psi (y\;|\;\theta )=\left\{\begin{array}{ll} -\theta , & \;y<-\theta \\ \;\theta , & \;y>\theta \\ \;y, & \;\text{otherwise.} \end{array}\right)$$

Here, *θ*>0 determines how extreme an observation must be to be relocated, as well as the replacement value. A common choice is *θ*=*τs*, where typically *τ*∈[1.5,3] and *s* is a robust estimate of the standard deviation (SD). A robust scale estimator is the Median Absolute Deviation (MAD), defined as the median of the values $\left|y_{j}-\hat{m}\right|$, where $\hat{m}$ is the median of *y*_1_,…,*y*_*p*_. For normally distributed observations, *s*_*M*_=1.4826·MAD corresponds to SD.

Winsorization of copy number data may be achieved by first estimating the trend in the data and then Winsorizing the residuals. Let the observations representing copy numbers in *p* genomic loci be **y**=(*y*_1_,…,*y*_*p*_), ordered according to genomic position. A simple estimator of the trend is the median filter. The trend estimate ${\hat{m}}_{j}$ in the *j*th locus is then given by the median of *y*_*j*−*k*_,…,*y*_*j* + *k *_for some *k*>0, e.g. *k*=25. The SD of the residuals $y_{j}-{\hat{m}}_{j}$ may then be estimated with the MAD estimator *s*_*M*_, and Winsorized observations $y_{1}^{w},\ldots ,y_{p}^{w}$ obtained by $y_{j}^{w}={\hat{m}}_{j}+\Psi (y_{j}-{\hat{m}}_{j}\;|\;\tau s_{M})$. Often, such simple and fast Winsorization is sufficient. However, copynumber also includes an iterative procedure with improved trend estimation based on the segmentation procedures described below (see Additional file 1).

### Single sample segmentation

Consider first the basic problem of obtaining individual segmentations for each of a number of samples. Suppose attention is restricted to one chromosome arm on one sample. For each of the *p* loci, the obtained measurement can be conceived of as a sum of two contributions:

(1) $$y_{j}=z_{j}+\varepsilon_{j}$$

where *z*_*j*_is an unknown parameter reflecting the actual amount of sample DNA at the j’th locus and *ε*_*j*_represents measurement noise. A breakpoint is said to occur between probe *j* and *j* + 1 if *z*_*j*_≠*z*_*j* + 1_. The sequence *z*_1_,…,*z*_*p*_ thus implies a segmentation *S*={*I*_1_,…,*I*_*M*_} of the chromosome arm, where *I*_1 _consists of the probes before the first breakpoint, *I*_2 _consists of the subsequent probes until the second breakpoint, and so on. To fit model (1), we minimize the penalized least squares criterion

(2) $$\overset{p}{\underset{j=1}{\sum }}{\left(y_{j}-z_{j}\right)}^{2}+\gamma \cdot |S|$$

with respect to the sequence *z*_1_,…,*z*_*p*_. Here, |*S*| denotes the number of segments in *S*, and *γ*>0 is a constant that controls the trade-off between seeking a good fit to the data (the first term) and restraining the number of level shifts (the second term). The minimizer ${\hat{z}}_{1},\ldots ,{\hat{z}}_{p}$ of (2) is fully determined by the segmentation *S*, since the best fit ${\hat{z}}_{j}$ on a given segment *I* is the average ${\bar{y}}_{I}$ of the observations on that segment. Substituting the latter into (2) we obtain the equivalent criterion:

(3) $$L(S\;|\;y,\gamma )=\underset{I\in S}{\sum }\underset{j\in I}{\sum }{\left(y_{j}-{\bar{y}}_{I}\right)}^{2}+\gamma \cdot |S|$$

(4) $$\;\;\;=\underset{I\in S}{\sum }\underset{j\in I}{\sum }y_{j}^{2}-\underset{I\in S}{\sum }{\left(\underset{j\in I}{\sum }y_{j}\right)}^{2}/n_{I}+\gamma \cdot |S|$$

where *n*_*I *_denotes the number of probes in segment *I*. Note that the first term in (4) does not depend on the segmentation *S*, hence minimization of (3) is equivalent to minimizing

(5) $$L^{\prime }(S\;|\;y,\gamma )=-\underset{I\in S}{\sum }{\left(\underset{j\in I}{\sum }y_{j}\right)}^{2}/n_{I}+\gamma \cdot |S|.$$

Naive optimization of the cost function (5) with respect to the segmentation *S* requires examination of every possible division of the probes on a chromosome arm into segments. For large *p*, this is not practically feasible. However, a much more efficient implementation based on dynamic programming and requiring only *O*(*p*^2^) operations is available. Dynamic programming is a method for solving complex problems by breaking them down into simpler subproblems, and specifically for problems where global decisions can be decomposed into a series of nested smaller decision problems. The crucial observation that allows the use of dynamic programming to solve the present segmentation problem is that the optimal segmentations on each side of a breakpoint are mutually independent. This can be used to iteratively build up a solution to the global segmentation problem. Suppose we know the optimal segmentations from the first probe up until the (*k*−1)st probe. Assume furthermore that the optimal segmentation for the *k* first probes contains breakpoints. Then the optimal segmentations from the last of these breakpoints and downwards has already been computed. Thus, by solving the above subproblems iteratively for increasing *k*, each step can utilize the results from the previous steps (see [24]). More formally, assume that the optimal segmentation of 1…*r* and the corresponding total error *e*_*r *_are known for all probes *r*<*k*. To extend the solution to *r*=*k*, first note that there must be a last segment starting at some index *j*≤*k*. From (5) we find that the cost term associated with that segment is:

$$d_{j}^{k}=\frac{1}{j-k-1}{\left(\overset{k}{\underset{r=j}{\sum }}y_{r}\right)}^{2}.$$

Then the total error for the optimal solution up until index *k* is found by minimizing the cost over the possible start positions *j* of the last segment. This cost consists of three terms: the cost of the last segment ($d_{j}^{k}$), the optimal cost of the segmentation up until that point (*e*_*j*−1_) and the penalty for the break point (*γ*):

$$e_{k}=\underset{j\in \{1,\ldots ,k\}}{\min}(d_{j}^{k}+e_{j-1}+\gamma )$$

where *e*_0_=0. The main work load of the above computation is to determine $d_{j}^{k}$ for all 1≤*j*≤*k*≤*p*. In interpreted languages (such as R) where loop execution is often quite inefficient, a considerable improvement of performance may be obtained by utilizing native-language vector operations. Let $a_{j}^{k}=\overset{k}{\underset{r=j}{\sum }}y_{r}$, $a_{k}=(a_{1}^{k},\ldots ,a_{k}^{k})$ and $d_{k}=(d_{1}^{k},\ldots ,d_{k}^{k})$. Then we may calculate all required coefficients through a simple recursion:

$$\begin{array}{lll} a_{k} & =[a_{k-1}\;\;0]+y_{k}\; & \;\\ d_{k} & =-a_{k}*a_{k}/(k:1)\; & \; \end{array}$$

where (*k*:1)=(*k*,*k*−1,…,1) and operators are vector-based. Hence, addition of a vector and a scalar adds the latter to each component of the former, and multiplications and divisions are performed component-wise on the operands, e.g., $a_{k}*a_{k}=[{\left(a_{1}^{k}\right)}^{2},\ldots ,{\left(a_{k}^{k}\right)}^{2}]$. Algorithm 1 summarizes the computations.

### Algorithm 1: Single sample PCF

*Input:* Log-transformed copy numbers *y*_1_,…,*y*_*p*_; penalty *γ*>0.*Output:* Segment start indices *s*_1_,…,*s*_*M *_and segment averages ${\bar{y}}_{1},\ldots ,{\bar{y}}_{M}$. 

1. Calculate scores by letting **a**_0_=[ ] and **e**_0_=0, and iterate for *k*=1…*p*:

• **a**_*k*_=[**a**_*k*−1_ 0] + *y*_*k*_

• **d**_*k*_=−**a**_*k*_∗**a**_*k*_/(*k*:1)

•$e_{k}=[e_{k-1}\;\;\min(d_{k}+e_{k-1}+\gamma )]$

storing also the index *t*_*k*_∈{1,2,…,*k*} at which the minimum in the last step is achieved.

2. Find segment start indices (right to left) $\left(s_{1}=t_{p},s_{2}=t_{s_{1}-1}\ldots ,s_{M}=1\right)$, where *M*≥1.

3. Find segment averages ${\bar{y}}_{m}=\text{ave}(y_{s_{m}},\ldots ,y_{s_{\left(m-1\right)}-1})$ for *m*=1,…,*M*, where *s*_0_=*p* + 1.

Throughout the paper we will tacitly assume that the penalty for the *i*th sample is $\gamma_{i}=\gamma {\hat{\sigma }}_{i}^{2}$, where ${\hat{\sigma }}_{i}^{2}$ is the estimated sample specific residual variance. In this way, we avoid scale dependency, and obtain consistent results for samples with equal signal-to-noise ratios. Such rescaling is also done by default in copynumber. Note that replacing the data $y_{j}^{i}$ for the *i*th sample with $y_{j}^{i}/{\hat{\sigma }}_{i}$ for *j*=1,…,*p*, and rescaling after estimation, has the same effect. In copynumber, the algorithm has also been extended to allow a constraint on the least number of probes in a segment.

### Multi-sample segmentation

Detection of very short or very low amplitude segments requires a small penalty *γ*, with low specificity as a potential result. However, when such segments are common to several samples, joint segmentation of multiple samples is an additional mechanism to increase sensitivity. This is a main motivation for introducing multi-sample segmentation methods that impose common breakpoints across all samples. Such methods are potentially useful for discovery of copy number variations (CNVs) and in those instances where the origin of the samples implies that segment boundaries are partly shared. Multi-sample segmentation with high penalty on breakpoints may also be used to obtain low-dimensional descriptions of the data, which may form the basis for defining variables to be used in statistical procedures relating aberration patterns to clinical outcome. In the following, we describe a direct generalization of single sample PCF to handle multiple samples simultaneously, obtaining common breakpoints for all the samples with minimal residual sum of squares for a given number of breakpoints. Suppose copy number measurements $y_{i}=(y_{1}^{i},\ldots ,y_{p}^{i})$ for samples *i*=1,2,…,*n*are obtained at the same loci in each sample. By direct generalization of the criterion (3), we seek in multi-sample PCF the minimizer of

(6) $$L(S\;|\;y_{1},\ldots ,y_{n},\gamma )=\overset{n}{\underset{i=1}{\sum }}L(S\;|\;y_{i},\gamma )$$

where *L*(*S* | **y**,*γ*) is defined as in (3) and *S* is a given segmentation common to all samples.

### Algorithm 2: Multi-sample PCF

*Input:* Log-transformed copy numbers for *n* samples **y**_1_,…,**y**_*p*_∈*R*^*n*^; penalty *γ*>0.*Output:* Common segment start indices *s*_1_,…,*s*_*M *_and segment averages ${\bar{y}}_{1},\ldots ,{\bar{y}}_{M}\in R^{n}$. 

1. Calculate scores by letting **A**_0_=[ ] and **e**_0_=0, and iterate for *k*=1…*p*:

• **A**_*k*_=[**A**_*k*−1_ 0] + **y**_*k*_

• $d_{k}=-1^{T}(A_{k}*A_{k})/(k:1)$

•$e_{k}=[e_{k-1}\;\;\min(d_{k}+e_{k-1}+\mathrm{n\gamma})]$

storing also the index *t*_*k*_∈{1,2,…,*k*} at which the minimum in the last step is achieved.

2. Find segment start indices (right to left) $\left(s_{1}=t_{p},s_{2}=t_{s_{1}-1}\ldots ,s_{M}=1\right)$, where *M*≥1.

3. Find segment averages ${\bar{y}}_{m}=\text{ave}(y_{s_{m}},\ldots ,y_{s_{\left(m-1\right)}-1})$ for *m*=1,…,*M*, where *s*_0_=*p* + 1.

The multi-sample PCF algorithm (see Algorithm 2) is in principle quite similar to single sample PCF. However, when updating the solution from *k*−1 to *k*, the sums and sums of squares for the segments must be accumulated and stored separately for each sample. This can still be done iteratively, implying that the computational effort will be approximately equal to carrying out single sample PCF on the same set of samples. Since the noise level may vary between samples, normalisation of the samples prior to segmentation and corresponding rescaling after estimation is advisable. It may also be desirable to scale the samples, e.g. to adjust for different tumor percentages. Thus, prior to running multi-sample PCF, we may replace **y**^*i*^ by $w_{i}y^{i}/{\hat{\sigma }}_{i}$ for *i*=1,…,*n*, where *w*_*i *_are weights and ${\hat{\sigma }}_{i}$ is an estimate of the SD. In copynumber normalization is performed by default for multi-sample PCF while further weighting is left as an option for the user.

### Allele-specific segmentation

The PCF algorithm is easily adapted to variants of the basic segmentation problem discussed above. Here, we consider an adaptation to handle SNP genotype data. We then have for each SNP locus a measurement of (total) copy number (logR) as well as the B allele frequency (BAF). We may also have measurements of copy number only for a number of additional loci. The B allele frequency is a number between 0 and 1 indicating the allelic imbalance of a SNP. For a homozygous locus we have BAF close to 0 or 1, while for a heterozygous locus with an equal number of the two alleles A and B, BAF will be close to 0.5. An imbalance between the number of A’s and B’s results in a BAF value deviating from 0.5. A change in the total number of copies of a segment will alter the logR value, hence result in a level shift in the logR track. Unless the copy number change is balanced with respect to the two alleles, the BAF value will also change. In cases involving multiple copy number events at the same locus, the change may manifest itself only in one of the two tracks. For example, a loss of one copy of A followed by a gain of one copy of B would lead to unchanged logR and changed BAF. The purpose of the allele-specific PCF algorithm is to detect breakpoints for all such events. It fits piecewise constant curves simultaneously to the logR and the BAF data, forcing breakpoints to occur at the same positions in both. We emphasize that the purpose of the allele-specific PCF algorithm is segmentation only and not to make allele-specific copy number calls. However, such calls can be made on the basis of the segmentation described below, and this is done e.g. in the ASCAT algorithm (Allele-Specific Copy number Analysis of Tumors) which estimates allele-specific copy numbers as well as the percentage of cells with aberrant DNA and the tumor ploidy [26]. Suppose the data are given by (*r*_*j*_*b*_*j*_) for *j*=1,…,*p*, where *r*_*j *_denotes the logR value and *b*_*j *_the BAF value at the *j*th locus. For copy number probes, only *r*_*j *_is given and *b*_*j *_will be missing (henceforth coded as NA). For germline homozygous probes, the BAF values are noninformative and should be omitted from the analysis. If the germline genotype is known (e.g. from a matching blood sample), the user should replace the corresponding BAF values by NA. If the genotype is not known, the algorithm will apply a proxy to handle this issue (see below). Prior to segmentation, the allele-specific PCF algorithm performs the following steps: 

The BAF data are mirrored around 0.5 by replacing *b*_*j*_with 1−*b*_*j*_if *b*_*j*_>0.5.

BAF values *b*_*j*_<*θ*are replaced by NA. By default *θ*=0.1. If germline homozygous probes have previously been replaced by NA’s, let *θ*=0.

Let ${\tilde{b}}_{1},\ldots ,{\tilde{b}}_{m}$ denote the nonmissing B allele frequencies. Corresponding copy number values ${\tilde{r}}_{1},\ldots ,{\tilde{r}}_{m}$ are found by pairing each logR probe with the nearest B-allele probe (ignoring those with missing values) and then averaging logR values paired to the same B-allele probe. Finally, let $y_{1}=({\tilde{b}}_{1},\ldots ,{\tilde{b}}_{m})$ and $y_{2}=({\tilde{r}}_{1},\ldots ,{\tilde{r}}_{m})$.

The remaining part of the allele-specific PCF algorithm is then essentially an adaptation of the multi-sample PCF algorithm applied to two samples. It finds a common segmentation *S* for the two tracks by minimizing the penalized criterion

(7) $$L(S\;|\;y_{1},y_{2},\gamma )=L(S\;|\;y_{1},\gamma )+L(S\;|\;y_{2},\gamma )$$

where *L*(*S* | ·,*γ*) is defined as in (3).

### Fast implementations of PCF

The PCF algorithms may be generalized to allow breakpoints only at certain prespecified positions. Combined with simple heuristics, this may be used to further enhance the computational speed of PCF. For brevity we describe only the single sample segmentation case here; however the copynumber package contains fast implementations of both single- and multi-sample PCF. Computationally inexpensive methods can be used to identify a set of potential breakpoints among which the breakpoints of the solution to (3) are highly likely to be found. Suppose we restrict our attention to such a set of potential breakpoints. All relevant information for solving the optimization problem in (3) may then be condensed into three arrays containing the number of observations between two potential breakpoints, the corresponding sum of the observations and the sum of squares. Based on these quantities, PCF may be used with straightforward modifications. Since the algorithm is of order *O*(*q*^2^), where *q* is the number of potential breakpoints, the potential increase in speed is substantial. Algorithm 3 outlines the procedure, while possible heuristics for finding potential breakpoints are discussed below. One way to identify potential breakpoints is to use high-pass filters, i.e. a filter obtaining high absolute values when passing over a breakpoint. The simplest such filter uses for each position *i* the difference $\overset{i+k}{\underset{j=i+\;1}{\sum }}y_{j}-\overset{i}{\underset{j=i\;-\;k\;+\;1}{\sum }}y_{j}$ for some *k*. To reduce artifacts due to the abrupt edges of such a filter, the copynumber implementation assigns half weight to the outer 1/3 of the observations on each side. Fast implementations of such filters in R may be obtained using the cumsum function. We currently use two filters with k=3 and 12, respectively; additionally the single sample PCF implementation includes a filter searching for aberrations of length equal to the lowest accepted one. These filters together identify about 15% of the probe positions as potential breakpoints. An additional way to speed up the computations on long sequences is to initially divide the sequence into overlapping subsequences, and iteratively find the solution.

Having found the solution for the *m* first subsequences, we use high-pass filters to detect potential breakpoints for subsequence *m* + 1, and then use the fast PCF algorithm with the latter potential breakpoints as well as those found by PCF on earlier subsequences. The intention behind this iterative approach is to reduce potential boundary effects. Due to the quadratic order of the algorithm, this division into subsequences implies a substantial efficiency gain. In copynumber, subsequences are used when the chromosomal arm length exceeds 15000 probes, with subsequences of length 5000 and overlap 1000.

### Algorithm 3: Fast PCF

*Input:* Log-transformed copy numbers *y*_1_,…,*y*_*p*_; penalty *γ*>0.*Output:* Segment start indices *s*_1_,…,*s*_*M *_and segment averages ${\bar{y}}_{1},\ldots ,{\bar{y}}_{M}$. 

1. Apply heuristics to find potential breakpoints *r*_0_,*r*_1_,…,*r*_*q*_, where *r*_0_=1 and *r*_*q*_=*p* + 1.

2. Form aggregates by letting $u_{k}=\overset{r_{k}-1}{\underset{j=r_{k-1}}{\sum }}y_{j}$, where *k*=1,…,*q*.

3. Calculate scores by letting **a**_0_=[ ], **c**_0_=[ ], **e**_0_=0, and iterate for *k*=1,…,*q*:

• **a**_*k*_=[**a**_*k*−1_ 0] + *u*_*k*_

• **c**_*k*_=[**c**_*k*−1_ 0] + *r*_*k*_−*r*_*k*−1_

• **d**_*k*_=−**a**_*k*_∗**a**_*k*_/**c**_*k*_

• $e_{k}=[e_{k-1}\;\;\min(d_{k}+e_{k-1}+\gamma )]$

storing also the index *t*_*k*_∈{1,2,…,*k*} at which the minimum in the last step is achieved.

4. Find segment start indices (right to left) $s_{1}=r_{t_{q}},s_{2}=r_{t_{s_{1}-1}}\ldots ,s_{M}=1$, where *M*≥1.

5. Find segment averages ${\bar{y}}_{m}=\text{ave}(y_{s_{m}},\ldots ,y_{s_{\left(m-1\right)}-1})$ for *m*=1,…,*M*, where *s*_0_=*p* + 1.

## Results and discussion

### Selection of penalty

**Figure 2:**
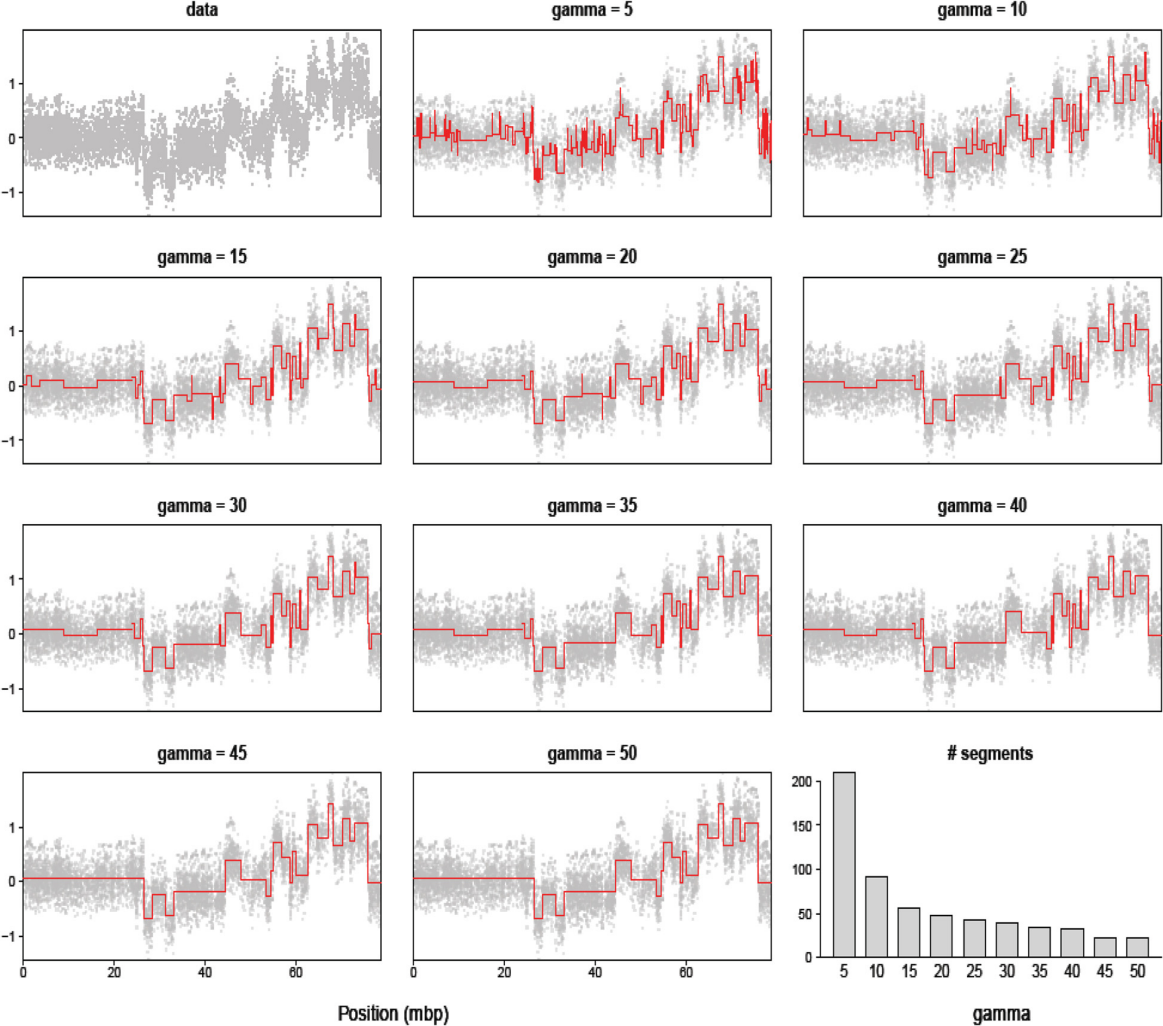
**The effect of changing the penalty *γ *in PCF.** The plot in the upper left corner shows the copy number data for a selected chromosome (in this case, chromosome 17), while the lower right plot shows the number of segments found by PCF as a function of *γ*. The remaining plots show the segmentation curves for ten different values of *γ*. The plot was created with the function plotGamma in copynumber.

The selection of parameters determining the trade-off between high sensitivity (i.e. few missed true aberrations) and high specificity (i.e. few false aberrations) is important in all segmentation procedures. In PCF, this is controlled by the single penalty parameter *γ*. A number of general model selection criteria exist, such as Cross-Validation, the Akaike Information Criterion (AIC) and the related Schwarz’s Bayesian Information Criterion (BIC). However, model selection for copy number segmentation is complicated by several factors. First, the distribution of the data at hand may vary substantially. An important example is the presence of local trends mimicking smaller aberrations; such low-amplitude “waves” in the data may e.g. be due to variations in GC-content (see, e.g., [9]). Second, the purpose of the analysis may favor either higher sensitivity or higher specificity. For example, in clinical studies aimed at finding prognostic markers, the main focus may be on the most pronounced and commonly occurring deviations, while detecting more sporadic aberrations may simply increase the noise level. In our experience, the above model selection criteria tend to give too small penalty estimates and thus undersmooth the data. This is consistent with previous investigations showing that AIC and BIC are not appropriate for the breakpoint problem (for details and discussions of other alternatives, see [6,27]). Simulation studies of specificity may suggest a lower bound on the penalty *γ*. For this purpose, sequences of independent and normally distributed observations without underlying aberrations were generated, and PCF was applied with different choices of *γ*. At *γ*=12 the number of falsely called aberrations is about 0.5 per 10.000 probes, at *γ*=10 roughly 2 per 10.000 probes, at *γ*=8 roughly 10 per 10.000 probes, and for *γ*≤6 the number of falsely called aberrations is substantial. This suggests *γ*≈8−12 as a lower bound. Since the number of false aberrations per chromosome increases with increasing probe density, low values are most relevant for arrays with low probe density. In the presence of local trends, the number of false calls tends to inflate and the penalty should thus be increased above the lower bound. A fairly conservative penalty of *γ*=40 is the default in the copynumber package. This provides a starting point for exploration of the best penalty value for the specific problem at hand, however a systematic inspection of results obtained for different penalties is advisable. Figure 2 illustrates the effect of changing *γ*. Notice that the main features in the data are captured across the whole range of *γ*-values, while finer details are only evident for smaller values.

### Aberration calling

**Figure 3:**
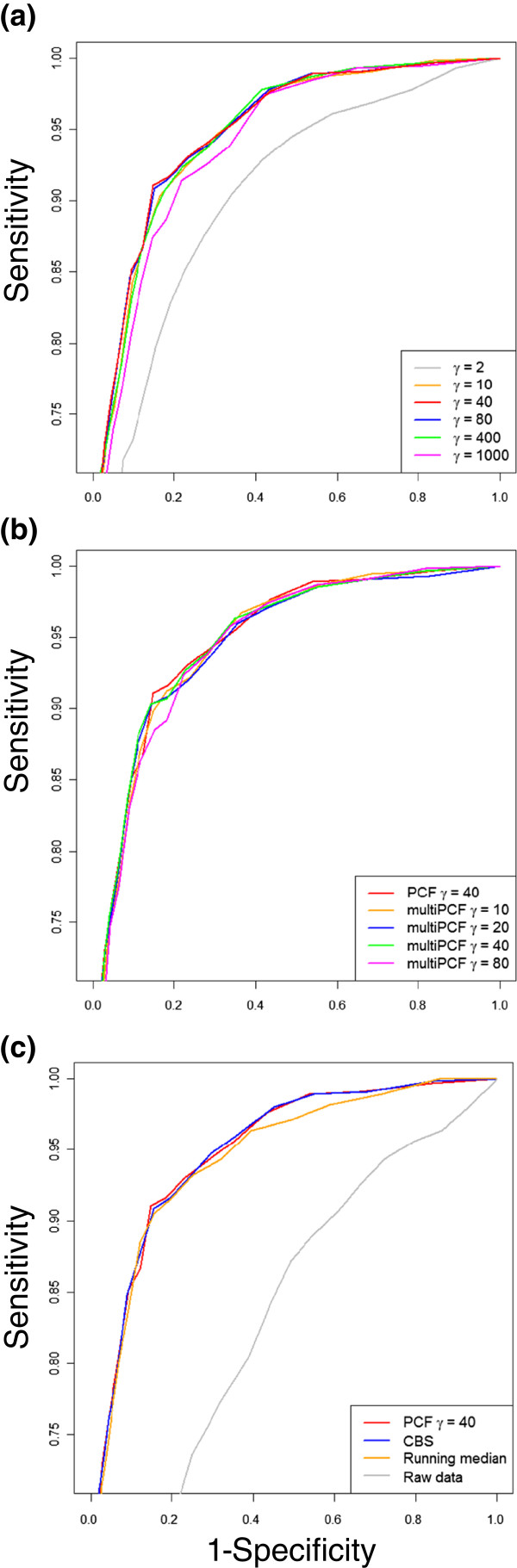
**Aberration calling accuracy.** The ROC-curves show the sensitivity and specificity for a sequence of thresholds as calculated by comparing aberration calls to the classifications made in a MLPA-analysis on the same data material. In panel **(a)**, classifications were made based on PCF segmentations found for a wide range of *γ*-values. Notably, the classification accuracy is not affected much by the choice of *γ*, except to some extent for very low values. Panel **(b)** shows that aberration calls based on multi-sample PCF segmentations are about as accurate as those based on single sample PCF. In panel **(c)**, ROC-curves are shown for calls made on the basis of the segmentations found by PCF and CBS, a running median with window size 50 and raw data. In terms of aberration calling accuracy, PCF and CBS give nearly the same results, while using the running median gives slightly less accurate classifications. Using only raw data leads to much poorer accuracy. Note the range on the ordinate axis.

Aberration calling is used for detection of recurring alterations and in many other analyses. Introducing a parameter *θ*>0 that determines the sensitivity of the aberration calling (and hence what to consider as biologically significant aberrations), we call probes for which ${\hat{z}}_{j}<-\theta$ as losses and probes for which ${\hat{z}}_{j}>\theta$ as gains. Optionally, different thresholds *θ*_ + _and *θ*_−_ may be used for gains and losses. To examine how well PCF aberration calling manages to distinguish between normal and aberrant regions, performance was compared with a very accurate measurement method. Specifically, aberration calls obtained with PCF on the basis of 1.8M SNP array data on 40 samples were compared with calls obtained with MLPA (Multiplex Ligation-dependent Probe Amplification; see Additional file 2 for details). Since MLPA is limited to a small set of genomic positions, only 88 loci were used for the comparison. In all samples combined, MLPA identified 546 aberrant and 2542 normal loci (the remaining 432 loci were ambiguous or unclassified and left out of the analysis). Using the MLPA-classification as the gold standard, the sensitivity and specificity of PCF aberration calling were calculated for a range of threshold values *θ*. Figure 3 shows the resulting ROC curves, and panel (a) illustrates how the results for PCF depend on the choice of *γ*. Importantly, aberration calling appears to be only moderately dependent on the choice of parameter values over a fairly wide range of *γ*-values.

### Single- versus multi-sample segmentation

**Figure 4:**
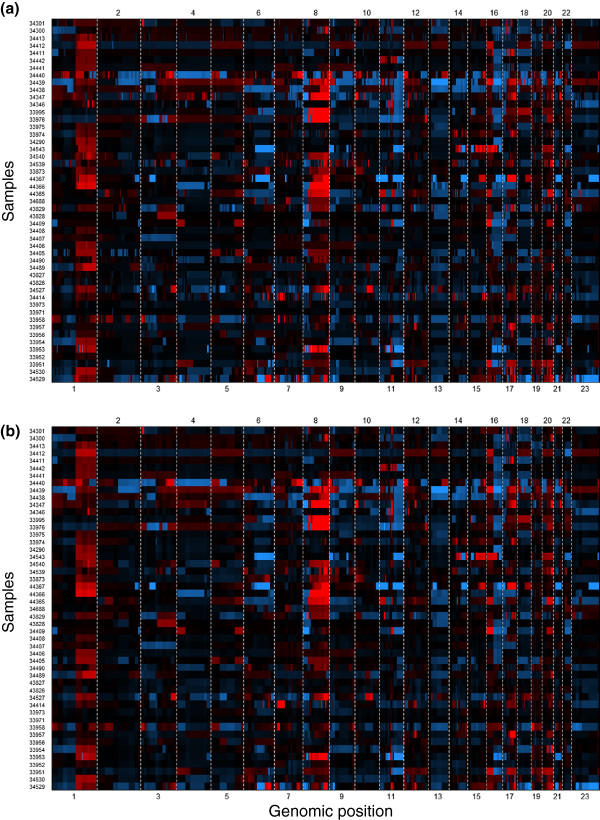
**Comparison of results from single sample and multi-sample PCF.** In single sample PCF, *γ*=40 was used, while in multi-sample PCF, *γ*=120 was used to limit the number of segments. Note that the estimated aberration patterns are quite similar; indicating that the multi-sample PCF estimates (panel **b**) should be well suited as variables in statistical analyses. On a more detailed level there are differences, e.g., longer segments in the single sample analysis (panel **a**) are divided into subsegments with slightly different estimates in the multi-sample analysis. The plot was created with the function plotHeatmap in copynumber.

Whether the initial segmentation of a dataset is most appropriately done using single- or multi-sample methods depends both on the purpose and the data. Using methods with common breakpoints for samples will increase the power for detecting concordant but quantitatively weak segments, while it will reduce the ability of detecting (or correctly positioning) discrepant breakpoints. A well known example of aberrations with common boundaries is germline copy number variants (CNVs), thus some proposed algorithms for CNV detection utilize segmentation with joint segment borders (e.g. [21]). Another important example of samples with (partially) common segment boundaries arises when the samples originate from different clones of the same (early) tumor. This is illustrated below in two examples, one on disseminated tumor cells from breast carcinomas, the other on tumor clones found at successive biopsies from lymphoma patients. Recent reports [28] on marked variations in aberration patterns within the same tumor is likely to increase the number of studies using several samples taken from each tumor. What is common as well as what differs in the aberration patterns will then be of interest, motivating the combined use of single- and multi-sample methods. In applications searching for genomic copy number *hot spots* with relevance to cancer development, it may be important to utilize the precise delineation of the aberrations found in each sample, and thus the use of single-sample methods is most appropriate. The identification of the relevant recurrent aberrations may then utilize post processing tools like GISTIC [29], KCsmart [30] or cghMCR [31] (see also the review in Rueda [18]). If focus is on clustering samples or on constructing regression variables for relating more broad aberrations to clinical outcome, one may consider using multi-sample methods. However, to be useful, the estimates from the multi-sample methods should in a proper way reflect the main information content in each sample. This implies that a multi-sample analysis should result in estimates approximating those obtained from single-sample analyses. Figure 4 shows heatmaps of results from single- and multi-sample PCF for 49 breast cancers from the so-called MicMa data set (see [32] and Additional file 2) analyzed on 244K Agilent arrays. The main features appear to be well reflected in the multi-sample analysis. On a more detailed level, differences can be observed: the multi-sample solution misses some short aberrations occurring in only a few samples, aberration borders are sometimes slightly shifted, and longer segments obtained with single sample PCF are often divided into subsegments with slightly different copy number estimates. The moderate difference between the results of single- and multi-sample PCF was also confirmed by a comparison of the ability to detect specific aberrations as revealed by comparison to MLPA analyses, see Figure 3b. This indicates that at least for cancer types where aberrations are focused in certain areas of the genome, methods using joint boundaries might be considered for constructing variables to be used in further statistical analysis.

### Comparing tracks: Analysis of disseminated tumor cells

**Figure 5:**
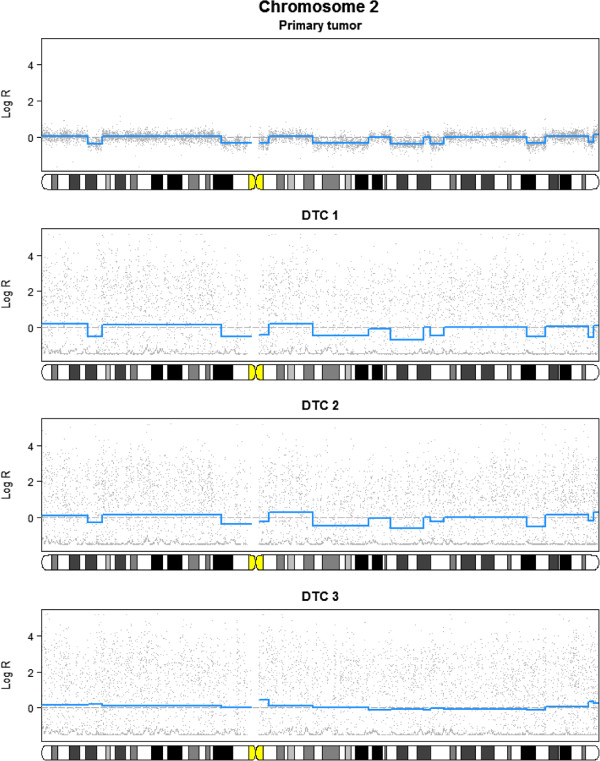
**Analysis of disseminated tumor cells (DTCs) with multi-sample PCF.** The top panel shows the primary tumor and the three panels below show single cells morphologically classified as DTCs (all for chromosome 2). High noise levels make separate analyses of each DTC difficult; co-analyzing multiple DTCs, possibly together with a primary tumor, thus facilitates an evaluation of the degree of correspondence between the aberration patterns. In the present case, two DTCs seem to have aberration patterns similar to the primary tumor, while the last cell has an essentially flat (balanced) pattern and is probably a hematopoietic cell misclassified as a DTC. The plot was created with the function plotChrom in copynumber.

Disseminated tumor cells (DTCs) are detected in the bone marrow of some patients with breast carcinomas. The presence of DTCs in the bone marrow identifies patients with less favorable outcome (see, e.g., [33]), and genomic characterization of such cells is of substantial interest. It is still an open question to what extent the aberration patterns in DTCs correspond to those found in the primary tumor; the DTCs may potentially have obtained new aberrations or, alternatively, the cells may have originated from (early) subclones of the tumor with less aberrations. It is possible to analyze single cells using aCGH; however, currently the noise level is high, making it difficult to draw definitive conclusions from a single cell. However, since segment boundaries are assumed to be partly common, we tested the multi-sample PCF algorithm on breast cancer cases from which DTCs were available (cf. [32] and GEO accession number GSE27574). Figure 5 shows the results on a set of DTCs and the corresponding primary tumor from one such patient. Since multi-sample PCF is used, segment boundaries are common, while the estimated level in each segment is determined by the individual DTC/primary tumor. In Figure 5, two of the single cells seem to have a pattern similar to the primary tumor. The last one has an essentially flat (balanced) profile and is likely to be a hematopoietic cell misclassified as a tumor cell (separation of DTCs from other cells is often difficult). These data thus indicate that the aberration pattern of the DTCs quite closely reflect that of the primary tumor. With only two single cells present, Figure 5 primarily shows that DTCs inherit the aberrations of the primary tumor; with higher numbers of cells, multi-sample PCF may also be used to search for aberrations found in DTCs but not in the tumor.

### Defining variables: Genetic evolution in follicular lymphoma

**Figure 6:**
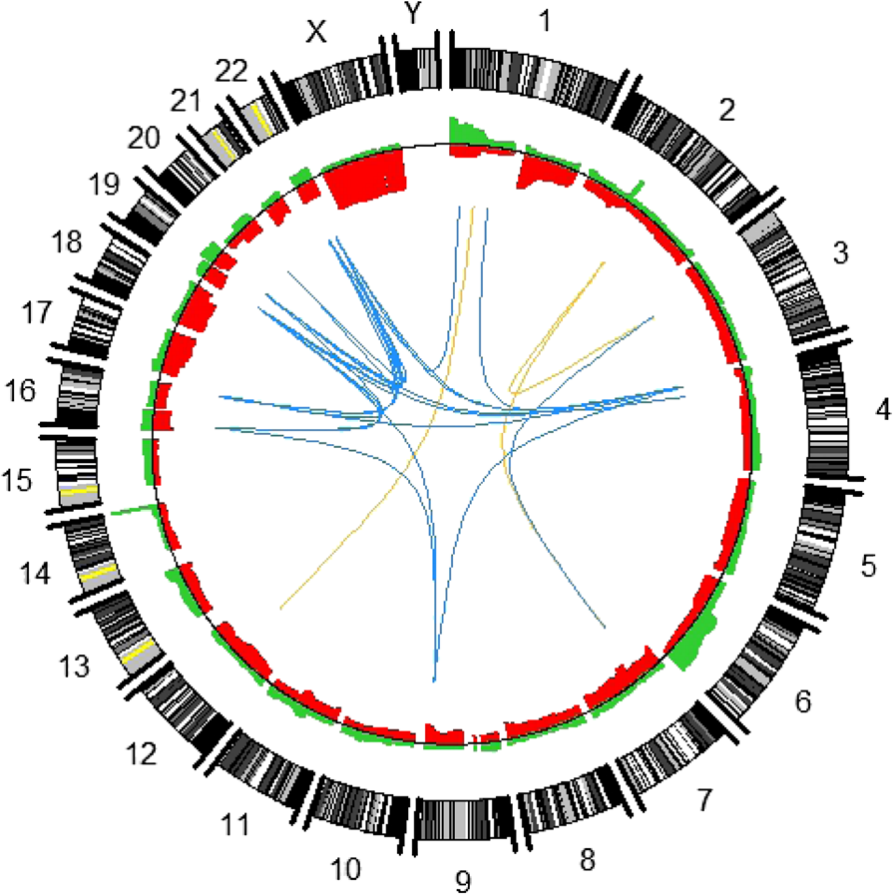
**Whole-genome view of aberrations in the follicular lymphoma data.** The plot is based on all 100 biopsies, and aberrations were defined as copy number estimates above 0.05 (for gains) or below -0.05 (for losses). Aberration frequencies are shown in red for gains and green for losses. Correlations between the copy number activity at different genomic locations are shown as arcs (blue for positive correlations and yellow for negative correlations), using a correlation threshold of ±0.68 to determine which correlations to display. Aberration frequencies are based on the segmentation found with single sample PCF (with *γ*=16 and *kmin*=3), while correlations are based on the segmentation found with multi-sample PCF (with *γ*=6). The plot was created with the function plotCircle in copynumber.

Follicular lymphoma is normally a slowly progressing malignancy, but relapses are common and the disease is usually fatal. In a recent study, 100 biopsies from 44 patients diagnosed with follicular lymphoma were evaluated using a custom-made aCGH platform consisting of 3k BAC/PAC probes [34]. A whole-genome view of aberration frequencies (based on single sample PCF) and highly correlated aberrations (based on multi-sample PCF) are shown in Figure 6.

Although the delineation of segments varied between biopsies, several areas with a high frequency of aberrations could be detected. To try to identify aberrations with prognostic potential, we therefore found a common segmentation for the initial biopsies taken from each of the 44 patients using the multi-sample PCF algorithm. Removing very low variance segments, 93 segments remained. The corresponding copy number estimates were used as covariates in a multivariate Cox proportional hazards regression. This revealed 11 segments for which gains were significantly associated with a survival disadvantage. A particularly strong association was detected for gains on chromosome X in male patients. To study the relation between successive biopsies taken from the same patient, multi-sample PCF was applied to each patient individually (see Additional file 2). As expected, many aberrations are common, but interestingly, some aberrations are present in early biopsies and not in later ones. This contradicts the hypothesis of linear development which states that late tumor clones arise from earlier ones, and supports the alternative hypothesis of parallel evolution in different lymph nodes.

### Allele-specific copy number analysis in breast cancer

**Figure 7:**
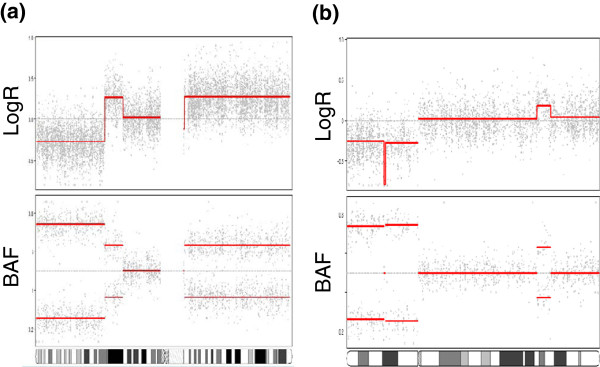
**Allele-specific PCF analysis of SNP array data.** Results are shown for a breast carcinoma sample in the MicMa cohort for chromosome 1 (panel **a**) and chromosome 17 (panel **b**). The points in the upper two panels show observed total copy numbers (logR) while the points in the lower two panels show observed B allele frequencies (BAF). The red curves show the result of applying the allele-specific PCF segmentation method to the data. The plot was created with the function plotAllele in copynumber.

Copy number alterations have been extensively studied in breast cancer. To what degree gains and losses are associated only with certain alleles has been less studied. In a recent study, genotyping of 112 breast carcinoma samples was performed using Illumina 109K SNP arrays, and the ASCAT method was used to infer the allele-specific copy numbers at each locus [26]. However, to do this we first had to segment the data; for this purpose we applied allele-specific PCF segmentation to all samples. In Figure 7, the result of this segmentation is shown for one particular sample and two different chromosomes. In Figure 7a, the segmentation of chromosome 1 is shown, and we clearly identify three segments on the p-arm with copy numbers less than two, larger than two, and identical to two (we assume here that tumor ploidy is 2). Suppose we consider only germline heterozygous loci, in which case the allelic ratio is 1/2 when no aberrations are present (one copy of B and two copies in total). The BAF track reveals allelic imbalance in the first two segments, and more pronounced in the first segment than in the second. This is consistent with a loss of one copy in the first segment (i.e. a hemizygous loss, resulting in an allelic ratio of 0/1 or 1/1 depending on whether the A-allele or the B allele is lost), and a gain of one copy in the second segment (resulting in an allelic ratio of 1/3 or 2/3 depending on which allele is gained). The third segment has an allelic ratio of 1/2. Notice that in case of allelic imbalance, the observed allele ratio is substantially closer to 0.5 than expected by the above theoretical ratios. This attenuation of the signal (which also affects the logR values) is due to technical issues like cross-hybridization, as well as the fact that in reality the tumor is a mixture of cells with normal DNA (two copies of each locus) and tumor cells with aberrant DNA. In Figure 7b we notice a sharp trough in the logR track on 17p, accompanied by an allelic ratio close to 0.5.

If for a certain SNP locus one allele is substantially more frequently gained than the other allele, one may hypothesize that the former allele is subject to a larger selective pressure to change copy number. This, in turn, may be an indication of different roles being played by the two alleles with respect to cancer progression and evolution, suggesting that loci subject to allelic skewness can be potential unique markers for breast cancer development. Even from a relatively small number of samples, probes with highly significant allelic skewness have been identified in a genome-wide statistical evaluation [26].

### Outliers and Winsorization

While least squares methods are often favored due to their optimality properties, they are also known to be sensitive to extreme observations. Thus, except if the purpose is to search for short aberrations of biological origins (CNVs), we advise the use of an outlier handling procedure. To evaluate the proposed Winsorization scheme, we first established a suitable way of simulating extreme observations. A classical way is to use “contaminated normals”, where the error distribution is a mixture of two normal distributions [35]. With probability 1−*α* the error is drawn from a distribution *N*(0,*σ*^2^), and with probability *α*from *N*(0,*d*^2^*σ*^2^), typically with *d*=3 and *α*=0.05. We compared the fraction of outliers in observed copy number data to the corresponding fractions in normals and contaminated normals, using MAD to estimate SDs. For the normal distribution, the fraction of observations outside 3 SD is 0.27% and outside 5 SD < 0.00001*%*, while these fractions for the 5% contaminated normal are 1.64% and 0.42%. For the Agilent 244K used on the MicMa dataset, the fractions were 1.89% and 0.59%, that is, slightly above the values for the contaminated normal. For the 44K Agilent and Illumina 109K applied to the same data, the percentages were slightly lower (3 SD: 1.41% and 1.18%; for 5 SD 0.29% and 0.11%), however still indicating that the 5% contaminated normal is an appropriate distribution when evaluating robustness of copy number assessment procedures. Inspection of data obtained by the 318K Illumina, 4 x 180K Agilent and Nimblegen 2.1M arrays also confirmed the existence of substantial amounts of outliers. The PCF algorithm was tested with and without Winsorization on simulated data with outliers (Table 1). Outliers in the contaminated distributions may cause the detection of short false aberrations; such spikes occurred roughly ten times per 1000 probes, as compared to less than two times per 1000 for uncontaminated data. Table 1 further shows that Winsorization efficiently reduces the number of falsely detected aberrations and make results for the contaminated distribution roughly equal to the ones for the normal distribution. In line with these observations, outliers tend to change the form of aberrations (their height and length), while Winsorization brings the distribution fairly close to the one found for normal data (data not shown).

**Table 1:** Outlier effects

**Type**	**Distribution**	**Sensitivity(%)**	**Specificity(%)**	**False aberrations (%)**
	Normal	79.5	96.5	0.15
A	Normal w/5% contam.	78.8	93.7	1.04
	Normal w/5% contam., Winsor.	78.1	96.0	0.13
	Normal	78.9	93.6	0.20
B	Normal w/5% contam.	77.8	90.6	1.06
	Normal w/5% contam., Winsor.	77.5	93.3	0.15

Shown is the effect of Winsorization on simulated data with outliers and artificial (low-amplitude) aberrations. Two types of aberrations are considered: (A) aberrations of height 1.5 and length 10 probes and (B) aberrations of height 1.0 and length 30. The contamination consists of normals with SD=3 and the MAD estimate of SD equals 1.0. Sensitivity is the percentage of amplified probes that are detected as amplified, while specificity is the percentage of non-amplified probes classified as such. The false aberration column gives the percentage of aberrations not covering the central part of the real amplifications.

Another way to avoid that a few extreme observations result in a segment is to impose a lower limit on the length (number of probes) of a segment. With a lower length limit of five probes, we found about twice as many false spikes as with Winsorization when adjusting *γ*to give equal sensitivity for true aberrations. Still, simulations indicate that a lower limit on segment length is valuable in combination with Winsorization. Note that outliers of biological origin will be more extreme if the technology has an inherent low noise level, as is the case, e.g., for BAC arrays and for high throughput sequencing. Thus, outliers are not a sign of inappropriate functioning of a technique, but a characteristic of the data requiring consideration in the analysis. In summary, copy number data tend to contain a high fraction of outliers. These outliers often induce false aberrations, but simple procedures like Winsorization will efficiently reduce these undesired effects.

### Computational performance

In R, using the vector based PCF implementation described in Algorithm 1 implies a substantial efficiency gain over loop based implementation, roughly a 10-20 times reduction in time requirements. The fast implementation of PCF (Algorithm 3) gives a further marked reduction in computing time. On the MicMa 244k dataset (longest arm ≈10000 probes), the implemented fast version is about 15 times faster than the exact one, and uses around 3.5 minutes to process the 49 samples (4 seconds per sample, see Table 2). The multi-sample method was slightly faster than the single sample version.

**Table 2:** Computational performance

**Method**	**R package**	**Agilent 244K**		**Illumina 1.1M**	
		**Raw data**	**Outliers removed**	**Raw data**	**Outliers removed**
PCF	copynumber	4 (0.2)	4 (0.2)	23 (0.7)	22 (0.4)
Fused Lasso	cghFLasso	5 (0.2)	5 (0.2)	97 (0.7)	99 (3.3)
CBS	DNAcopy	15 (4.7)	35 (4.1)	71 (12.9)	219 (12.8)

The average computation time (in seconds) per sample is shown for copynumber (PCF), DNAcopy v1.30.0 (CBS) and cghFLasso v0.2-1 (Fused Lasso) on the MicMa 244 K data set (49 samples) and on the logR values from an Illumina 1.1 M SNP array data set (6 samples). The IQR over samples is given in parenthesis. The methods were applied to both raw data and data with outliers removed using the Winsorization method. All tests were performed on a PC with a 2.93GHz Intel i7 CPU with 8 Gb of memory running Windows 7 and R 2.15.1 (64-bit).

The deviations between the solutions found by the exact PCF and fast PCF on the MicMa set were small; in terms of reduction in variance (difference between sample variance and residual variance after fitting PCF curves) below 0.01%. The differences observed for the curves were typically small shifts in the border of aberrations. Thus, we conclude that the results from the fast procedure for practical purposes may be regarded as global solutions to (3), and the fast version is therefore used by default in copynumber. We also compared the performance of PCF with two other segmentation methods: Circular Binary Segmentation (CBS) [4,36] and Fused Lasso Regression (FL) [12]. In comparison studies [5,8], CBS has shown good performance in terms of sensitivity and false discovery rate. It is probably the most commonly used freely available algorithm and is also implemented in several commercial analysis tools. CBS is available in the R package DNAcopy, which is used for this comparison. FL is a more recent proposal implemented in the R package cghFLasso, and is one of three preferred methods in the web-based segmentation tool CGHweb [37]. Using default parameter settings, we compared the computing times of PCF, CBS and FL on the 49 samples in the 244 K MicMa data set, and on 6 samples from a 1.1 M Illumina SNP array (using the logR values). Table 2 gives the average computation time (in seconds) per sample. With no preprocessing of the data, PCF is on average 3-4 times faster than CBS on both data sets, and about 4 times faster than FL on the largest data set. Note that copynumber detects and operates on chromosome arms, while DNAcopy operates on whole chromosomes. This partly explains the difference in performance between PCF and CBS for the MicMa data set; for the Illumina data this has little impact due to the iterative approach used in PCF for the longest sequences. PCF was also markedly faster in evaluations based on simulated data; however, comparisons are complicated by the fact that the speed of CBS depends on the data in a nontrivial manner. As seen from the IQRs listed in parentheses in Table 2, the speed of CBS is quite variable from sample to sample while PCF and FL is nearly constant. Moreover, the table shows that CBS runs 2-3 times slower when outliers have been removed using Winsorization, underlining that the performance of CBS is highly data dependent. We underline that the above-mentioned results only relate to the current R implementations. As mentioned in the introduction, PCF is conceptually similar to the CGHseg method described by Picard et al.[6], and we also examined the computational performance of this method using the implementation in the R package cghseg. Using the version of CGHseg that requires a prespecified number of segments for each chromosome, the algorithm is fast, although the speed depends on the number of segments. Using the full CGHseg algorithm that automatically determines the number of segments, the algorithm is very slow for high-resolution data. Hence, making a fair comparison between PCF and CGHseg is difficult.

### Segmentation accuracy

We further compare the accuracy of the segmentation solutions found by PCF and CBS. Figure 3c shows ROC curves using MLPA classifications as the truth, and then applying a range of aberration calling thresholds to PCF estimates, CBS estimates, a running median with window size 50 and raw copy number data (details in Additional file 2). Results for PCF and CBS are similar, both achieving high sensitivity and specificity. The running median also gives good results, illustrating that many probes are fairly easy to classify and that the gain obtained by using methods like CBS and PCF is mainly an improved classification close to borders between segments. We also repeated the simulation study in [8] where CBS was found to be the most sensitive method while also having the lowest false discovery rate. Again, we found that PCF and CBS had very similar performance (results not shown). A more detailed comparison of segmentation results shows that overall results are quite similar for single sample PCF and CBS, however for both methods the results depend on the choice of parameter values and the handling of extreme observations, see Additional file 3. In conclusion, PCF and CBS typically provide similar results and have equivalent accuracy when parameters are tuned appropriately.

## Conclusions

Copy number segmentation based on least squares principles and combined with a suitable penalization scheme is appealing, since the solution will be optimal in a least squares sense for a given number of breakpoints. We have proposed a suite of platform independent algorithms based on this principle for independent as well as joint segmentation of copy number data. The algorithms perform similarly as other leading segmentation methods in terms of sensitivity and specificity. Furthermore, the proposed algorithms are easy to generalize and are computationally very efficient also on high-resolution data. The Bioconductor package copynumber offers a user-friendly interface to the proposed algorithms.

Several extensions and modifications of the proposed least-squares framework are possible. In principle, the L2-based distance measure used in the current implementation of PCF is easily extended to general Lp-distances. However the current implementation is highly optimized for L2, and other distance measures would require substantial heuristics to obtain comparable computational performance. Another extension is to introduce locus specific penalties for breakpoints, thus essentially introducing a prior on the location of breakpoints. Work in progress includes specialized routines to handle high throughput sequencing data more efficiently and joint analysis of multiple samples in allele-specific PCF.

## Availability and requirements

**Project name:** Copynumber

**Project home page:**http://heim.ifi.uio.no/bioinf/Projects/Copynumber/

**Operating system(s):** All systems supporting the R environment

**Programming language:** R

**Other requirements:** No

**License:** GNU Artistic License 2.0.

## Abbreviations

aCGH: Array Comparative Genomic Hybridization; AIC: Akaike’s Information Criterion. ASCAT: Allele-Specific Copy number Analysis of Tumors; BAC: Bacterial Artificial Chromosome; BAF: B-Allele Frequency; BIC: Schwarz’s Bayesian Information Criterion; CBS: Circular Binary Segmentation; CNV: Copy Number Variation; DTC: Disseminated Tumor Cells; FL: Fused Lasso; HTS: High-Throughput Sequencing; IQR: Interquartile Range; MAD: Median Absolute Deviation; MLPA: Multiplex Ligation-dependent Probe Amplification; PCF: Piecewise Constant Fitting (the method used for segmentation in this paper); ROC: Receiver Operating Characteristic curve; SNP: Single-nucleotide Polymorphism.

## Competing interests

The authors declare that they have no competing interests.

## Authors’ contributions

The study was initiated by KL, ALBD and OCL. GN, KL and OCL drafted the manuscript. The software was written by GN with contributions from KL based on algorithms developed by GN, KL and OCL. PVL, HKMV, MBE and LOB contributed with examples and in discussions of the manuscript and software. OMR, SFC, RR and CC provided and analysed the MLPA data. All authors have read, commented on and accepted the final manuscript.

## Supplementary Material

Additional file 1 This pdf-file contains a formal description of the iterative PCF-based Winsorization algorithm. Click here for file

Additional file 2 This pdf-file contains a description of the three data sets used in this paper. Click here for file

Additional file 3 This pdf-file describes a comparison of segmentations performed by CBS and PCF on a MicMa sample. Click here for file
