# Supplementary material for: Copynumber: Efficient algorithms for single- and multi-track copy number segmentation
Source: BMC Genomics. 2012 Nov 4;13:591. doi: 10.1186/1471-2164-13-591 (PMC3582591; doi:10.1186/1471-2164-13-591)
Supplement: Additional file 1 — This pdf-file contains a formal description of the iterative PCF-based Winsorization algorithm. [file 1471-2164-13-591-S1.pdf]

Additional File 1

Iterative Winsorization to handle outliers

Winsorization is a common way of handling outliers, and works by modifying extreme observations to make them conform better to the distribution of other observations. Here, we describe a Winsorization algorithm that iteratively refines the detection of outliers and the assessment of copy number changes. The input to the algorithm is a sequence of copy number values (ordered according to their genomic location), and the output is a sequence of the same length consisting of the Winsorized data.

A median filter is used to obtain an initial trend estimate. Thereafter, robustness will be less critical and we can use the (less robust) PCF method. The iteration in step 2 leads to more precise estimates, but improvements are sometimes small. Thus, from a practical point of view, we may choose R to be small. Outliers are defined as observations for which $y_i \neq y_i^{\omega}$.

Algorithm: Iterative Winsorization

Input: Copy number data $y_1, \dots, y_p$ and $\tau > 0$.
Output: Winsorized copy number data $y_1^{\omega}, \dots, y_p^{\omega}$.

1. Obtain an initial trend $\hat{m}_{0,1}, \dots \hat{m}_{0,p}$ by applying a median filter with a window that includes k points on each side of the probe (default is $k = 25$).

2. For $r = 1, \dots, R$:

   - Calculate the SD of the residuals $y_i - \hat{m}_{r-1,i}$ using the MAD estimator $s_r = \hat{\sigma}_M$.
   - Calculate Winsorized estimates $y_{r,i}^{\omega} = \hat{m}_{r-1,i} + \Psi(y_i - \hat{m}_{r-1,i} \mid \tau s_r)$.
   - Calculate the trend $\hat{m}_{r,1}, \dots, \hat{m}_{r,p}$ of the data $y_{r,1}^{\omega}, \dots, y_{r,p}^{\omega}$ using Algorithm 1.

3. Final Winsorized observations are $y_i^{\omega} = y_{R,i}^{\omega}$ for $i = 1, \dots, p$.
