# Supplementary material for: Copynumber: Efficient algorithms for single- and multi-track copy number segmentation
Source: BMC Genomics. 2012 Nov 4;13:591. doi: 10.1186/1471-2164-13-591 (PMC3582591; doi:10.1186/1471-2164-13-591)
Supplement: Additional file 2 — This pdf-file contains a description of the three data sets used in this paper. [file 1471-2164-13-591-S2.pdf]

Additional File 2

Description of data sets

MLPA data set

A dataset of 40 primary breast carcinomas from the METABRIC cohort (Curtis et al. 2012) was used for evaluation of aberration calling. Two different platforms were selected to measure copy number status: SNP arrays and MLPA (Multiplex Ligation-dependent Probe Amplification).

MLPA measurements were obtained for 88 selected genomic loci. The copy number status of each sample for each separate locus was then classified as *normal*, *gain*, *amplified*, *loss of heterozygosity (LOH)*, *homozygous loss*, or *ambiguous* using Coffalyser (www.mlpa.com); 26 adjacent matched normals were used to normalise the tumour calls. For all samples combined, a total of 2542 probe values were classified as normal, and 546 probe values were classified as either *gain*, *amplified*, *loss of heterozygosity* or *homozygous loss*. The remaining 432 values were ambiguous or unclassified, and were left out of the subsequent analysis.

Corresponding CGH data were obtained using Affymetrix Genome-Wide Human SNP Array 6.0 as previously described (Curtis et al. 2012). This platform provides copy number ratios for ~1.8 M unique genomic loci. Aberration calling was performed by first segmenting the data and then scoring the absolute values of segments covering the positions of the 88 MLPA probes, using thresholds in the range 0 to 5 (with steps of 0.025). In the case of aberration calling based on a running median and raw data, the absolute value of the probe closest to the MLPA probe was used for the analysis.

Figure A shows the location and the log2-value of the 88 MLPA probes on top of the SNP array data for one sample.

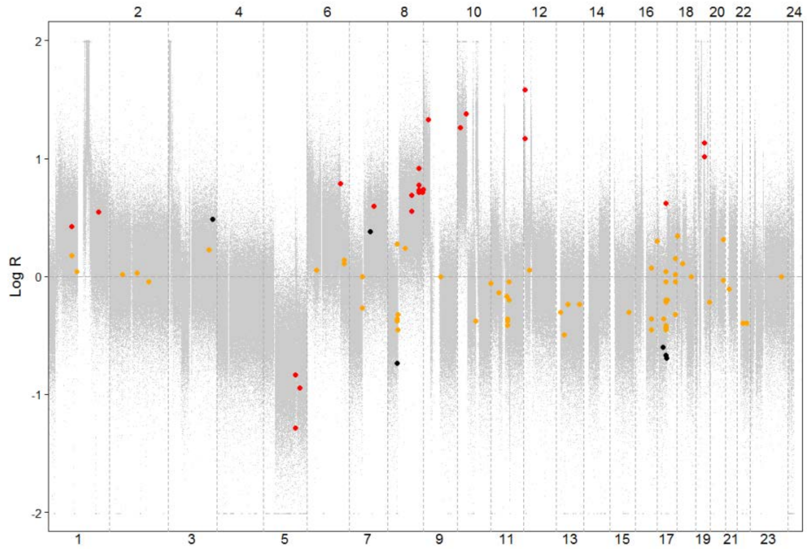

Figure A. Location and log2-value of the 88 MLPA probes on top of the SNP-array data for one sample. Probes classified as aberrations, normal and ambiguous by the MLPA analysis are shown in red, orange and black, respectively.

MicMa data set

The biomaterial from early breast cancers was collected from patients included in the Oslo Micrometastasis (MicMa) Study – Oslo1 (Wiedswang et al. 2003). The samples are a subset from a larger cohort of stage I-II primary breast tumors collected in 1995-98 at five hospitals in Norway (Ullevål University Hospital, Norwegian Radium Hospital, Aker University Hospital, Bærum Hospital and Buskerud Hospital, the three first now part of Oslo University Hospital). DNA from a subset of 49 tumor samples from primary surgery (47 primary tumors and 2 lymph node metastases) were analysed analyzed using Agilent's Human Genome CGH 244k Microarrays (Agilent Technologies, Santa Clara, California, USA) (Mathiesen et al 2011). These samples were used in Figures 2 and 4 in the paper, and also when comparing computational performance.

From a subset of patients, bone marrow mononuclear cells were available, and single cell array comparative genomic hybridization was performed for these cells (Mathiesen et al 2011). The bone marrow samples were collected at the time of surgery by aspiration from from posterior or anterior iliac crests. Potential disseminated tumor cells (DTCs) were detected by cytochemistry, as described previously (Wiedswang et al. 2003). These cases were the basis for the disseminated tumor cells example in the main text, including Figure 5.

For many single cells, a fraction of the probe values were very low. To avoid too strong influence of these probes in the copy number estimation, a lowest detectable value was defined and all values below this limit were set equal to the limit (typically -2 on the log2 scale). Following this procedure, the zero line was redefined to give an average value of 0 across all chromosomes for each cell. It was furthermore observed that data from single DTCs showed systematic fluctuations in probe values, probably due to the amplification process. These fluctuations were similar across all cells, including the blood cells. To avoid false aberrations due to these fluctuations, an initial step in the estimation was to construct a moving average based on available blood cells. For each probe value from the DTCs, the corresponding moving average value was subtracted, and the resulting residuals were used in the copy number estimation.

Follicular lymphoma data set

Samples from follicular lymphomas (FL) were selected from the archives of the Pathology Clinic at The Norwegian Radium Hospital, Oslo University Hospital, Norway. The selection criteria were the diagnosis of FL according to the WHO criteria and the presence of fresh frozen tissue from multiple samples of FL positive for the translocation t(14,18). A total of 100 samples from 44 patients (median age 44, range 29-71) diagnosed between 1987 and 2005 were selected; from 39 patients two or more successive biopsies were available. Median observation time was 88 months (range 10-294). The tumor cell content was above 50% in all samples (except one sample with 40% tumor cells).

DNA was isolated from frozen tissue. Copy number alterations (CNAs) were examined by array comparative genomic hybridization on in-house arrays containing approximately 4,500

BAC/PACs in quadruplicate at the resolution of 1Mb. The construction and preparation of the microarrays as well as the procedures for DNA-labeling and hybridization have been previously described (Meza-Zepeda et al. 2006). Arrays were scanned using the Agilent G2565BA scanner. Images were segmented and raw data were filtered in GenePix Pro 6.0. The whole dataset is available in the ArrayExpress database (www.ebi.ac.uk/arrayexpress, accession no. E-TABM-930).

As stated in the paper, a central aim of the study was comparison of aberration patterns seen in biopsies taken at successive relapses in the same patient. Figure B below shows whole-genome copy number estimates from three successive biopsies. Note the apparent loss of aberrations from an early to a late biopsy (see, e.g., chromosome 7q and 13). This indicates that the disease develops in parallel in different lymph nodes, as opposed to the late biopsies being direct descendants from the earlier ones.

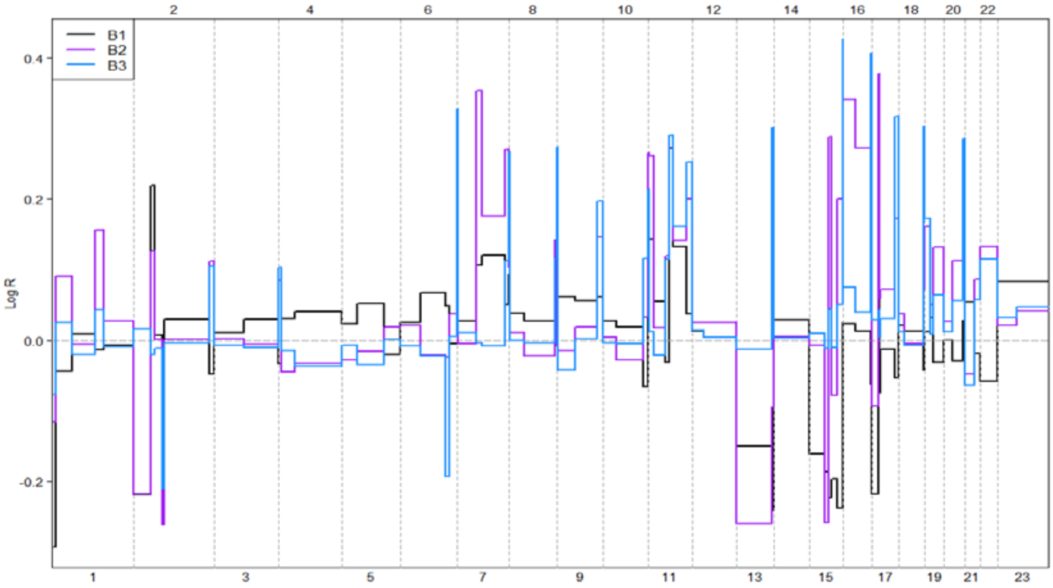

Figure B. Whole-genome copy number estimates from three successive biopsies from the same patient. The graph was created with the function *plotGenome* in *copynumber*.

References

Curtis C, Shah SP, Chin SF et al. The genomic and transcriptomic architecture of 2,000 breast tumours reveals novel subgroups. Nature 2012. doi:10.1038/nature10983.

Mathiesen RR, Fjelldal R, Liestøl K et al. High resolution analysis of copy number changes in disseminated tumor cells of patients with breast cancer. Int J Cancer 2011. doi:10.1002/ijc.26444.

Meza-Zepeda LA, Kresse SH, Barragan-Polania AH, et al. Array comparative genomic hybridizationreveals distinct DNA copy number differences between gastrointestinal stromal tumors and leiomyosarcomas. Cancer Res. 2006;66(18): 8984-8993.

Wiedswang G, Borgen E, Kaaresen R, et al. Detection of isolated tumor cells in bone marrow is an independent prognostic factor in breast cancer. J Clin Oncol 2003, 21: 3469-3478.
