# Supplementary material for: Copynumber: Efficient algorithms for single- and multi-track copy number segmentation
Source: BMC Genomics. 2012 Nov 4;13:591. doi: 10.1186/1471-2164-13-591 (PMC3582591; doi:10.1186/1471-2164-13-591)
Supplement: Additional file 3 — This pdf-file describes a comparison of segmentations performed by CBS and PCF on a MicMa sample. [file 1471-2164-13-591-S3.pdf]

Additional File 3

Comparison of PCF and CBS segmentations

The figures below compare segmentation results obtained with PCF and CBS. Winsorized copy number data for chromosome 5 from the sample MicMa-066 are shown. PCF segmentation is shown in red and CBS segmentation in blue.

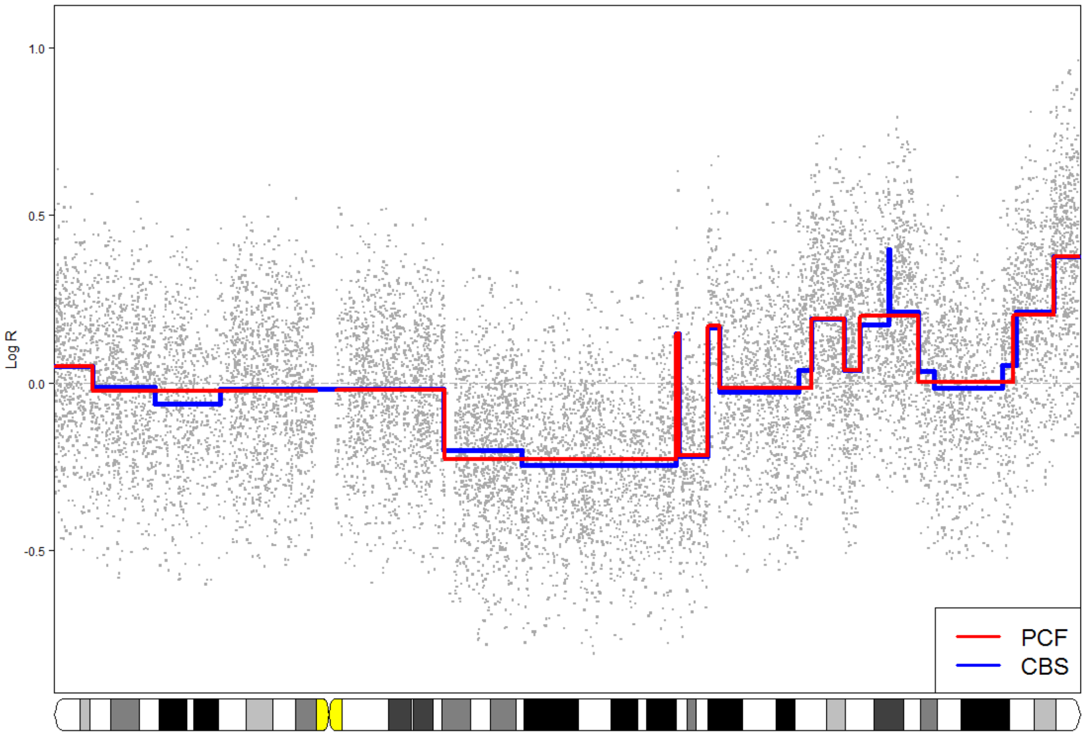

Figure A. PCF and CBS with default parameter values (except that min.width=5 in CBS to match the corresponding parameter kmin=5 in PCF). Overall features are the same. CBS detects some smaller local trends in the data and a narrow peak on 5q, while the default parameter setting in PCF ($\gamma = 40$) emphasises such local patterns in the aberrations less.

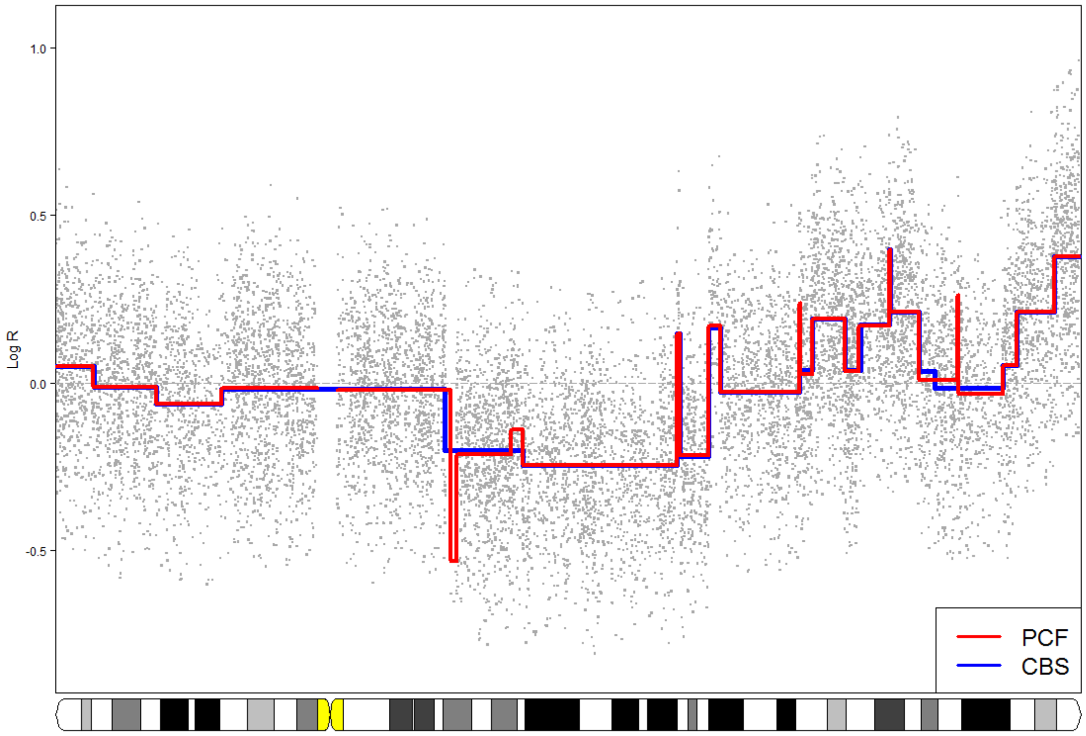

Figure B. PCF with $\gamma = 12$ and CBS with the same parameters as in Figure A. PCF now finds two additional narrow peaks on 5q.

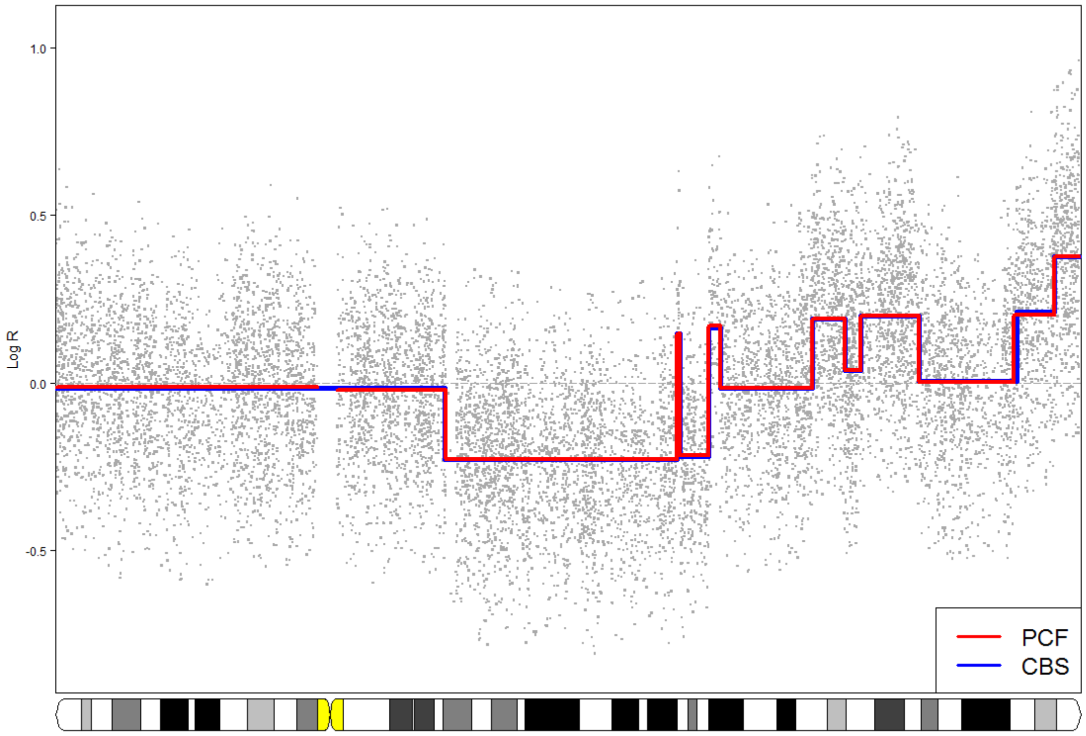

Figure C. PCF with kmin=5 and $\gamma = 60$ and CBS with min.width=5, $\alpha = 0.05$ (default is 0.1), undo.splits = ``sdundo'' (default is ``none'') and undo.SD = 0.5 (default is 3). Here PCF and CBS give practically identical segmentation results.
